# Fig Paste

**Published:** 1883-11

**Authors:** 


### FIG PASTE.

MANY persons suppose that "fig paste," or, as it is often called, "genuine Turkish fig paste," is made of figs; nothing of the sort.

An old friend of ours, who was an extensive manufacturer of candies in New York, and who has made tons of "fig paste," gave us the following recipe: Take 12 lbs. of wheat starch, and 100 lbs. of "A" sugar, or in that proportion; to that amount of sugar add half an ounce to one ounce of acetic acid, after the sugar is dissolved in sufficient water to thoroughly dissolve it; add to the starch enough water to thoroughly wet it up, and add it to the dissolved sugar; boil over coal fire in a *copper kettle* till done; then turn out in pans or moulds greased with beef suet. Stir constantly from the time the starch is put in till it is taken off the fire, or it will burn. Use a sharp-edged wood paddle to stir with. It must be thoroughly cooked, or it will soon become dry and hard. When cooked enough it remains soft, flexible and tough for months. Lemon or other flavoring may be stirred in just after it is taken off of the fire.

The above might be reduced down to the following proportions, and the "fig paste" made in a preserving kettle: Half pound of starch, 4 pounds of "A" sugar, one teaspoonful of acetic acid or vinegar. Flavor with a few drops essence of vanilla.

INSECT POWDER or "Persian powder," if fresh and good, will destroy cockroaches and croton bugs inevitably. It should be *puffed* into the crevices between the boards and under the sink, where they are usually found. If this is repeated every evening for a week there will be no more living bugs visible for a long time. Should this plan fail you may be certain the insect powder is not of the best quality, or that it has deteriorated.

